# 

**Published:** 1847-10-01

**Authors:** 


### ADDRESS TO THE READER.

The Readers of the Medico-Chirugical Review will perceive, by the Prospectus which accompanies the present Number, that it has been determined to incorporate with it, in the future publication, The British and Foreign Medical Review, hitherto conducted by Dr. Forbes. By this arrangement the Proprietors have the gratification of being able to state, that the services of the most valued Contributors to both of these Reviews will be combined—and that there is every reason to believe that the result will be the production of a work second to none in Europe for the soundness, depth, and variety of its Medical and Chirurgical investigations.

London,

*September* 30, 1847.

### BIBLIOGRAPHICAL RECORD.

1. The Retrospect of Medicine; being a Half-yearly Journal, containing a Retrospective View of every Discovery and Practical Improvement in the Medical Science. Edited by *W. Braithwaite.* Part 15. Jan.—June, 1847. 8vo, pp. 483. London.

2. The Half-yearly Abstract of the Medical Sciences; being a Practical and Analytical Digest of the Contents of the principal British and Continental Medical Works published in the preceding Six Months. Edited by *W. H. Ranking,* M.D. Vol. V. Jan.—June, 1847. 8vo, pp. 424. London.

3. The Medical Examiner and Record of Medical Science. Edited by *Robert M. Huston,* M.D. No. 29—30, May and June, 1847. Philadelphia.

4. An Experimental Inquiry into the Functions of the Great Sympathetic Nerve. By *C. Radclyffe Hall,* M.D. Part I. 8vo, pp. 126. Plates. London, 1847.

5. A Guide to the Use of the Buxton Waters. By *William Henry Robertson,* M.D. Fourth Edition, revised. Foolscap 8vo, pp. 32. London, 1847.

6. A Copy of Reports on Sir *William Burnett's* Disinfecting Fluid. Ordered by the House of Commons to be printed, 20th July, 1847. Pp. 14.

7. Cholera, Dysentery, and Fever, pathologically and practically considered; or the Nature, Causes, Connexion, and Treatment of these Diseases in all their forms. By *Charles Searle,* M.D. 8vo, pp. 140. London, 1847.

8. A Treatise on Diet and Regimen. By *William H. Robertson,* M.D. Fourth Edition, Part III., 8vo. London, 1847.

9. Twenty-seventh Annual Report of the Directors of the Dundee Royal Asylum for Lunatics, submitted, in Terms of their Charter, to a General Meeting of the Directors, 21st June, 1847. With the Report of the Medical Officers. 8vo, pp. 62. Dundee, 1847.

10. Seventeenth Annual Report of the Belfast District Asylum for the Insane Poor of the Counties of Antrim and Down, and of the Town of Carricfergus. For the Year ending 31st March, 1847. Drawn up by the Resident Physician. 8vo, pp. 43. Belfast.

11. A Letter to Benjamin Rotch, Esq., Chairman of the Committee of Visitors; on the Plan and Government of the Additional Lunatic Asylum for the County of Middlesex about to be erected at Colney Hatch. By *John Conolly,* M.D. 8vo, pp. 27. London, 1847.

12. The Consciousness of Right and Wrong a just Test of the Plea of partial Insanity in Criminal Cases. Illustrated by the Case of William Stalker, indicted at the Cumberland Lent Assizes, 1847, for the wilful Murder of his Wife. By *C. Lockhart Robinson,* M.D. 8vo, pp. 18. Edinburgh, 1847.

13. Proceedings of the National Medical Conventions, held in New York May 1846, and in Philadelphia May 1847. 8vo, pp. 175. Philadelphia, 1847.

14. The American Journal of the Medical Sciences. Edited by *Isaac Hays,* M.D. 8vo, pp. 284. July, 1847. Philadelphia.

15. The Human Brain; its Structure, Physiology, and Diseases, with a Description of the Typical Forms of Brain in the Animal Kingdom. By *Samuel Solly.* Second Edition. 8vo. pp. 684. London, 1847.

16. On the Causes and Treatment of Abortion and Sterility; being the Result of an extended Practical Inquiry into the Physiological and Morbid Conditions of the Uterus, with reference especially to Leucorrhœal Affections, and the Diseases of Menstruation. By *James Whitehead,* F.R.C.S. 8vo, pp. 426. London, 1847.

17. The Microscopic Anatomy of the Human Body in Health and Disease. Illustrated with numerous Drawings in Colour. By *Arthur Hill Hassall.* Parts 10 and 11. London, 1847.

18. An Account of a Simple Means of Moderating the Effects of Fire upon the Human Body. By Mr. *F. A. Bulley,* F.R.C.S., Surgeon to the Royal Berkshire Hospital, Reading. [Reprinted from the Medical Times.]

*This paper gives an account of the benefit its author has derived from the employment of Treacle diluted with three parts of water, at a temp. of* 98°, *as an application to Burns of various degrees of intensity. Lint soaked in the mixture is to be kept constantly applied to the part, renewing morning and evening, and moistening at intervals.*

19. On the Use of Nitrate of Silver in the Cure of Erysipelas. By *John Higginbottom.* F.R.C.S. E. Nottingham. (Read before the Provincial Medical and Surgical Association.)

*The Profession is much indebted to Mr. Higginbottom for having many years since called its attention to the valuable properties of the Nitrate of Silver, and especially to its power in arresting the progress of Erysipelas. In the present communication he recommends the application at a far earlier period of the disease than he formerly deemed advisable; such mode of using it, with attention to the digestive organs, often rapidly cutting short its progress. He employs the following solution:—Arg. Nitr. four scruples, Nitric Acid six drops, Distilled Water four drachms, previously well washing the part first with soap and water, and then with pure water. So used, it is especially beneficial in erysipelas, threatening to spread over the scalp, to which it may be freely applied without inducing vesication.*

20. The Preservation of Infants in Delivery. Being an Exposition of the Chief Cause of Mortality of Still-born Children. By *Richard King,* M.D., M.R.C.S. 8vo, pp. 60. London, 1847.

21. The Chemistry of Vegetable and Animal Physiology. By Dr. *G. J. Mulder*. Translated from the Dutch, by Dr. *P. F. H. Fromberg*. With an Introduction and Notes, by *J. F. W. Johnstone*, F.R.S.L. & E. Part III. 8vo, pp. 267. Eight coloured Lithographs. Edin. 1847.

22. Contributions to the Pathology and Treatment of the Scorbutus which is at present prevalent in various parts of Scotland. By *Charles Ritchie*, M.D. (From the Monthly Journal of Medical Science.)

*Dr. Ritchie has here drawn up a very excellent account of this formidable disease as it has prevailed in Scotland—in common with entire Europe—during the late Spring. For reasons stated in our Periscope we cannot however agree with him in referring its production exclusively to defective diet, exposure to severe weather and the like; and we are among those whom he speaks of as "turning away from what is known and tangible, to seek the causes of the epidemic in the unknown and impalpable obscurities of an aerial constitution."*

23. Anecdota Sydenhamiana: Medical Notes and Observations. By *Thomas Sydenham*; hitherto unpublished. Second edition, pp. 80. Oxford, 1847.

*We are glad to observe that these "Notes, &c." of a cotemporary and acquaintance of Sydenham himself have reached a second edition. Our readers are probably aware that the profession is indebted to Dr. Greenhill of Oxford for the possession of this little work. As a matter of course, all the members of the Sydenham Society should have a copy of it.*

24. On the Duties of Physicians, resulting from the Physician. By the late Rev. *Thomas Gisborne*, M.A. Pp. 56. Oxford, 1847.

*A re-print of a portion of the author's well-known and useful work, "Enquiry into the Duties of Men in the higher and middle classes of Society in Great Britain, resulting from their respective Stations, Professions, and Employments," 6th Edit.* 1811. *We all stand in need of being reminded of our duties towards those who commit their health, and often their happiness too, to our keeping. To those beginning the practice of the medical profession, much of the advice in these pages may be truly useful.*

25. A few Remarks on the Expectant Treatment of Diseases. By ΑΚΕΣΤΗΣ. Pp. 16.

*These remarks are intended as a defence of "legitimate medicine," and in answer to certain recent heresies in a cotemporary Journal on the subject of what has been called the "natural method" of treating diseases. We cannot congratulate the author on the success of his attempt. Much of what he says is altogether beside his argument, and the tendency of some of his observations is altogether very objectionable.*

26. Unhealthiness of London, and the Necessity of Remedial Measures. By *Hector Gavin*, M.D., F.R.C.S.E. Pp. 70. London, 1847.

*Contains much useful information respecting the state of health in the metropolis, the fearful amount of disease that might be prevented, and the simple and efficient means of attaining this most desirable object. The lecture is exceedingly well adapted for a popular audience*

27. Consumption of the Lungs and Asthma, arrested and cured, in the Majority of Cases, by Inhalation and other Rational Means. By *Daniel Carr*, M.D. 12mo, pp. 200. London, 1847.

*A work that is altogether discreditable to the writer (whose address is Birmingham), if he be a regularly-educated member of the profession. What shall we say of an M.D., who appends "a series of questions on Consumption and Asthma, which will enable patients, who are desirous of consulting a physician, to state their symptoms clearly, either personally or by letter?" Need we say more respecting the style and purport of the book?*

28. Theorie des Neuro-viscerites ou Fievres Primitives. Par *Ant. Hugon*. 8vo, pp. 111. Paris, 1847.

*The author is a decided anti-Broussaist, and an energetic advocate of the essentiality of fevers. His views are generally sound and practical, and we have derived much pleasure from the perusal.*

29. Gazette Medicale, July to September.
*In exchange.*

30. L'Union Medicale.
*In exchange.*

31. Dublin Medical Review.
*In exchange.*

32. Edinburgh Medical and Surgical Journal for July.
*In exchange.*

33. British and Foreign Medical Review for July.
*In exchange.*

34. Edinburgh Monthly Journal of Medical Science, for July, August, and September.
*In exchange.*

---

ERRATUM.

Dr. E. Kennedy's paper, referred to in page 405, appeared in the number of the Dublin Medical Journal for February, not May, last.

### INDEX.

Abdomen, wounds of, Guthrie on.... 88
Abdomen, wounds of, sutures in .... 91
Abdomen, effects of blows on the.... 95
Abdomen, treatment of injuries of .. 97
Abortion, habitual, Assafœtida in .. 266
Abortion, habitual, induction of premature labour in ............ 545
Abortion and Sterility, Whitehead on 377
Abortion, statistics and causes of.. .. 395
Accidental Productions............ 40
Acid, Hydrocyanic, Nunneley's experiments on.................... 116
Acid, Hydrocyanic, *modus operandi* of 118
Acid, Hydrocyanic, treatment of poisoning by .................... 120
Africa, Bryson's Report on Diseases of ....... .............. 431, 449
Aged, frequency of pulse in ...... .. 523
Albuminuria in Pregnancy ........ .. 277
Albuminuria induced by blisters .... 540
Alkalis, Mialhe on................ 552
Alum, Mialhe on the action of...... 554
Amputation, employment of the Saw in 372
Amputation, Results of............ 373
Amygdalæ, extirpation of.......... 51
Anæmia, cerebral ................ 297
Analysis, insufficiency of ultimate.... 407
Aneurism, liability of various arteries to 45
Aneurism, predisposing and exciting causes of.. .................. 45
Aneurism, Bellingham on Compression in.......................... 46
Animals, Experiments on .......... 518
Anus, Artificial .................. 95
Aorta, Inflammation of the ........ 43
Apoplexy, Grisolle on ............ 34
Apothecaries, Guy Patin on the .... 175
Army-Surgeons, Douglas on the Claims of .................... 245
Arsenic, Poisoning by ............ 36
Arsenic, employment of, in diseases of the skin... ............... 247, 267
Arteries, deposits in the coats of .... 44
Arteritis, Crisp on................ 43
Arteritis, Chronic diffused.......... 257
Arthritis Puerperal ............... 555
Asylums, Lunatic, Conolly on...... 128
Asylums, Lunatic, improved state of 416
Auscultation, Depaul on Obstetrical.. 495
Barber-Surgeons, Guy Patin on .... 177
Bellingham on Compression in Aneurism ........................ 46
Bischoff on the Ovum ............ 5
Bladder, Wounds of the .......... 96
Bladder, Inflammation of, from Blisters 540
Blisters, Rules for the application of.. 371
Blisters, Induction of Strangury by.. 540
Blisters, Mialhe on the composition of 542
Blisters, effect of on the Blood...... 325
Blood, Polli's Researches on the .... 303
Blood, formation of the buffy coat of 303
Blood, density of the.......... 306, 321
Blood, causes of the coagulation of the 308
Blood, Plethoric and Anæmic ...... 309
Blood, Period of Coagulation of the.. 310
Blood, condition of, in inflammation 314
Blood, coagulation within the body.. 461
Blood, effects produced on the, by bleeding .......... .......... 319
Bloodletting, Guy Patin on ........ 172
Bloodletting from the Jugular Vein .. 252
Bloodletting, Tanchou on........ .. 279
Bloodletting, Regulation of ........ 310
Bloodletting, effects of, on the mass of the blood...................... 319
Bloodletting, indications and contra-indications of............... 320, 323
Bloodletting, formation of dropsy after 325
Bloodletting, habitual ............ 325
Bloodletting, effect of excessive .... 325
Blood-vessels, Crisp on the diseases of 42
Bone, Owen on the Structure of .... 156
Bone, Owen's description of a ...... 158
Bone, Flourens on the formation of.. 426
Bowman's Physiological Anatomy .. 326
Brain, Solly on the Human ........ 285
Brain, mode of study of the ........ 289
Brain, Physiology of the .. ....... 293
Brain, Ramollissement of the ...... 299
Brain, Nature of Congestion of the .. 301
Breast, question of removing for cancer ............................ 370
Breast, Velpeau on Abscess of the .. 543
Brookes on Inhalation of Ether...... 246
Bryson, Report on Diseases of African Station ...................... 431
Burnett's Disinfecting Fluid........ 483
Burns, treatment of .............. 256
Callus, formation of .............. 430
Cancer, Question of operating for.... 370
Cardialgia, Seymour on............ 98
Catamenia, Nature of the .......... 379
Catamenia, age at appearance of .... 382
Cells, Mulder on animal and vegetable 414
Cerebellum, Relation of, to the Spinal Cord ...................... 291
Chancre, Marchal on .............. 57
Chelius' System of Surgery ........ 361
Chemistry, Animal, Liebig on the Study of, .................... 405
Chemists, Guy Patin on .......... 173
Children, bleeding from the jugular vein 252
Children, Trousseau on the dentition of 275
Children, Still Born .............. 522
Cholera, Andral on the intestinal secretions in ...................... 544

Cholera, treatment of ............ 517
Christ, Physical Death of...... 453, 468
Ciliary Muscle, Todd and Bowman on 330
Circumcision, Jewish mode of performance of......... .............. 373
Cochlea, Stricture of the .......... 331
Cod-Liver-oil, substitute for........ 282
Collyria, Mialhe on astringent ...... 554
Coma, Anæmic, Solly on .......... 297
Conception, Time and Seat of ...... 8
Congelation, Shrimpton on ........ 52
Conolly on Lunatic Asylums........ 128
Cookery, Liebig on the Rationale of 410
Copland on the Yellow Fever........ 183
Cornea, Structure of the .......... 329
Corpus Callosum, formation of the .. 293
Cranium, Owen on the homologies of 521
Crisp on the Blood-vessels.......... 42
Crosse on Inversio Uteri .......... 124
Croup, Alum emetics in .......... 282
Crucifixion, nature and effects of the punishment of ................ 465
Curvatures, Chelius on............. 375
Cysts, formation of ....... ...... 38
Decidua, formation of the.......... 12
Delivery, state of the Vulva after .... 532
Dentition, Trousseau on .......... 275
Depaul, Auscultation, Obstetricale .. 495
Deshon on Cold and Consumption .. 357
Development, Owen on the laws of .. 154
Disease in relation to Sex .......... 250
Disinfecting Fluids, nature of ...... 483
Duncan on Medical Statistics ...... 250
Dysentery, Harty on .............. 69
Dysentery, Simple ............... 73
Dysentery combined with Fevers .... 75
Dysentery, the, of ancient London .. 83
Dysentery, infectiousness of ........ 85
Electricity, influence of, in the production of disease.................. 531
Embryology, importance of the study 2
Encephalon, comparative anatomy of 287
Epididymitis, Treatment of ........ 60
Epilepsy, Moreau on treatment of.... 256
Epilepsy, Solly on Pathology of .... 300
Epilepsy, Solly on treatment of .... 302
Epilepsy Complicated with Insanity.. 424
Ether, Inhalation of .............. 246
Exostosis, Roux on .............. 262
Eye, Foreign bodies, removal of, from 274
Eye, Sichel on preservation of...... 278
Eye, Irrigation in Diseases of the.... 548
Femur, fracture of the neck of...... 137
Fever, Typhoid, Grisolle on ........ 25
Fever, Typhoid, Serres on Æthiops mineral in ..................... 549
Fever, Typhus, Grisolle on ........ 28
Fever, Eruptive, Grisolle on........ 30
Fever, Eruptive, Serres on ........ 549
Fever, Intermittent, Grisolle on .... 31
Fever, Malignant Remittent........ 193
Fever, the Yellow, Copland on. .... 183
Fever, Yellow, analogy to other fevers 190
Fever, the Yellow, remittence of .... 199
Fever, the Yellow, question of a second attack ......................... 201
Fever, the Yellow, etiology of ...... 205
Fever, Yellow, epidemic visitation of 208
Fever, Yellow, influence of sanitary improvements on the............ 213
Fever, Yellow, at Boa Vista ........ 219
Fever, Yellow, McWilliam on the .. 218
Fever, Yellow, Quarantine and Preventive measures against ........ 233
Fever, Yellow, of Africa........ 435, 449
Fever, Yellow, Diarrhœa a precursor of 445
Fever, Yellow, treatment of........
Fever, the Eclair ............ 217, 447
Fibrine, conditions of, in the blood in inflammation .................. 314
Fishes, Owen on the Skeleton of .... 166
Flesh, Liebig on the juices of ...... 407
Flourens on the formation of Bone .. 426
Fœtus, Auscultation of the ........ 501
Frost-bites, Treatment of .......... 52
Ganglia, Hall on Functions of the.... 509
Gangrene, Dry, Crisp on .......... 42
Gleet, treatment of ............... 55
Gonorrhœa, treatment of, by injections 53
Gout, Seymour on................ 112
Gout, Skin disease in subjects of .... 105
Gray's Supplement to Pharmacopœia 283
Gream on Diet of Children ........ 251
Green on Mental Dynamics ........ 235
Grisolle Pathologie Interne ........ 22
Guthrie on Wounds of the Abdomen 88
Hæmorrhages, Grisolle on ........ 33
Hæmorrhages, Treatment by bleeding 325
Hall on the Sympathetic Nerve .... 506
Harty on Dysentery .............. 69
Hassall's Microscopic Anatomy...... 522
Heart, Influence of strong Emotions on the .......................... 455
Heart, Rupture of the ............ 459
Heart, Beau on the capacity of the cavities of the................... 534
Heart, Size of Cavities during arterial murmurs...................... 535
Heart, Effects of Age on the size of the 536
Heart, Polypi of the .............. 40
Heart, Abscess of the ............ 122
Hernia Humoralis, treatment of .... 60
Hernia, Ventral .................. 90
Hernia, Stricture of the intestine in.. 533
Hip-joint, chronic rheumatism of.... 142
Humerus, fracture of, near the shoulder 147
Hunt on Diseases of the Skin ...... 247
Hunterian Oration, Green's ........ 235
Hydrocele, radical cure of.......... 369
Hypochondriasis, Seymour on ...... 113
Impregnation, Bischoff on.......... 6
Infection, Sedillot on purulent...... 49

Inflammation, condition of the fibrine of the blood in ............314, 317
Insanity, Seymour on opium in .... 109
Insanity, Puerperal .............. 111
Insanity, Prevention of............ 136
Insanity, Study of ................ 136
Insanity, Exercise and Recreations in 130
Insane, Employments of the........ 131
Insane, Superintendence of ........ 132
Insanity, Religious Service in ...... 134
Insanity, Establishments for ........ 135
Insanity in Reference to Phrenology.. 296
Insanity, dependence of, on disease of the Brain.......... .......... 299
Insanity, Shakespeare's Delineations of 333
Insanity, Brigham on Medical Treatment of ...................... 339
Insanity, State of the Pulse in...... 340
Insanity, Shape of the Head in...... 341
Insanity, Hereditary Predisposition to........................ 341, 538
Insanity, Education of the predisposed to...... ...................... 342
Insanity, Homicidal .. ........... 342
Insanity, Plea of......... ......... 344
Insanity, Establishment for at Gheel 346
Insanity, Brierre on the English Asylums for ...................... 347
Insanity, Brierre on non-restraint treatment of ....... ......... 349
Insanity, improved condition of the establishments for ............. 417
Insanity not a protection from creditors 418
Insanity, Treatment of, at English Asylums ....................... 421
Insanity, combination with Epilepsy 424
Insanity, combination with General Paralysis ...................... 424
Insanity, moral treatment of........ 425
Insanity, Statistics of Royer-Collard on the ........................ 538
Intention, Union by the First ...... 50
Intercostal Neuralgia and Neuritis.... 259
Intestine, Wounds of the .......... 92
Iodine, Inhalation of.............. 352
Iris, Structure and Connections of the 329
Iris, Hall on the motions of the .... 508
Itch, Devergie on the.............. 269
Jaw, Lower the, Dislocation of...... 150
Jaw, Lower, Chronic Rheumatism of 151
Jugular vein, Bleeding from the...... 252
King on Still-born Children ........ 522
Kreatine and Kreatinine, Liebig on .. 407
Labour, Premature, Induction of.... 545
Lead, Anæsthesia from ............. 282
Ledoyen's Disinfecting Fluid........ 483
Leucorrhœa in relation to Abortion.. 397
Levy on Measles in the Adult ...... 477
Liebig's Researches on the Chemistry of Food ........................ 405
Lithotomy, Treatment before and after 364
Lithotomy compared to Lithotrity 366, 539
Lithotomy, Statistics of.........368, 539
Lithotrity, Civiale on............... 539
Liver, Wounds of the ............ 95
Lonsdale on Curvature of Spine .... 570
Lunacy, Report of Commissioners of 415
Lunatics, improved condition of .... 417
Lunatics, imprisonment of, for debt.. 418
Macilwain on Vivisection .......... 518
McWilliam's Report on Yellow Fever 218
Malaria, Nature of................. 516
Malgaigne on Duties of a Surgeon .. 254
Mania, Treatment of............... 421
Mania, Sutherland on the use of anodynes in .......................... 422
Measles of Adult, Levy on the...... 477
Meat, mode of boiling ............ 410
Meat, effects of salting ........... 412
Medicines disguised by Coffee ...... 555
Melancholia, Treatment of..... 112, 423
Melancholia, connexion with Phthisis, 113
Menorrhagia, Whitehead on........ 385
Menses, Properties and Source of ... 379
Menstruation, Spurious ........... 380
Menstruation, age of.............. 382
Menstruation, influence of factory labour on ...................... 383
Menstruation, period of terminating, 386
Menstruation, diseases succeeding .. 387
Menstruation during pregnancy .... 393
Microscope, importance of, in Morbid Anatomy ....................... 398
Mouth, the, in man and animals .... 249
Mulder, Chemistry of Vegetable and Animal Physiology ............. 405
Myopia, Sichel on .... ............ 278
Natural History, Green on ........ 236
Nerve, Sympathetic, Swan on the ... 237
Nerve, Sympathetic, Hall on the.... 506
Nervous System, Bell's Researches on the......................... 249
Nervous System, Solly on the ...... 294
Neuralgia, Intercostal ............ 259
Novellis on Scorbutus ............ 61
Operations, Surgical, Chelius ...... 361
Operations, Surgical, After-treatment of.......................... 362
Ophthalmia, Purulent of Infants .... 547
Ovarian Cysts, teeth in............ 38
Ovarian Cysts, serous ............ 39
Ovariotomy, Southam on.. ........ 121
Ovum, Structure of the ........... 3
Ovum, Bischoff's Researches on .... 5
Ovum, Development of............ 9
Owen's Lectures on Comparative Anatomy ...................... 153
Owen on the Vertebrate Skeleton ... 520
Paralysis, General, of the Insane .... 424
Paraphymosis, Reduction of ....... 374
Patin, Guy, Lettres de ............ 169
Perception, Nature of ............. 327

Pericardium, Effusion of Blood into.. 463
Periosteum, Agency in the formation of Bone .................... 427
Pharmacopœia, Gray's Supplement to, 283
Phrenology illustrated by Insanity . . . 296
Phthisis in relation to Sex ......... 250
Phthisis, Scudamore on Inhalation of, 351
Phthisis, Increase of Animal Heat .. 355
Phthisis, Evolution of Carbonic Acid. 356
Phymosis, Circumcision in ........ 373
Placenta, Structure of the ......... 13
Placenta, Hunter ................ 17
Placenta, Vessels of the............ 18
Pneumonia of the Aged ........... 281
Poisons, Action of, Grisolle on ..... 35
Poisons, limitation of the sale of .... 115
Polli, Researches on the Blood...... 303
Pompholyx in gouty habits ........ 105
Pregnancy, Use of Assafœtida in.... 266
Pregnancy, Albuminaria in ........ 277
Pregnancy, Auscultation in ........ 495
Pregnancy, Signs of ..........391, 532
Pregnancy, Menstruation during .. .. 393
Psoriasis in Gouty Habits........... 105
Puberty, female, influence of factory labour on ..................... 383
Pulmonary Consump. Scudamore on 351
Pulse, frequency of in the aged ..... 523
Pupil, Action of the ............... 507
Pyrosis, Seymour on............... 98
Radius, Fracture of lower end of the, 143
Ramolissement, microscopic examination of......................... 298
Receuil de Memoires de Medecine.... 49
Respiration, frequency of, in the aged, 523
Rete Mirabile, The ............... 288
Retina, Structure of the ............ 330
Sacrum, Eschar over the .......... 537
Sciatica, Seymour on ............. 106
Scorbutus, Novellis on ............ 61
Scorbutus, Chronic or Apyretic .... 63
Scorbutus, Pathology of ........... 63
Scorbutus, Acute ................. 64
Scorbutus, Complication .......... 65
Scorbutus, Treatment ............ 67
Scorbutus, State of the Bood in .... 525
Scorbutus, Andral on Fibrine in .... 528
Scorbutus, analogy with Typhoid .. 529
Scudamore on Pul. Consumption .. 351
Searle on Cholera ................. 516
Sensation, nature of .............. 540
Sex, influence of diseases according to, 250
Seymour, Thoughts on various Diseases 97
Shakespeare's Delineation of Insanity, 333
Shaw on Bell's Researches ......... 249
Shoulder, Congenital Dislocation.... 149
Sierra Leone, Topog. and Diseases of 432
Skeleton, Owen on the development of 156
Skeleton, Homological & teleological structure ..................... 158
Skin, Arsenic in diseases of the ..... 247
Skin, Treatment of Squamous Diseases of the ....................... 267
Small-pox, Ectrotic Treatment ..... 256
Smith on Fractures near Joints ..... 137
Solly on the Human Brain ........ 285
Soup, Liebig's formula for ......... 411
South's Household Surgery ........ 240
South's Translation of Chelius...... 360
Southam on Ovariotomy .......... 121
Spinal Cord, Structure of.......... 290
Spinal Cord, relation to Brain ...... 291
Spine, Lonsdale on Curvature of .... 510
Spleen, wounds of the ............ 96
Stallard on Abscess of the Heart.... 123
Statistics, Medical, Duncan on ..... 250
Sterility, Whitehead on Cause and Treatment of .................. 401
Stomach, Wounds of.............. 95
Stomach, Pain of the.............. 98
Stomach, Ulceration of the ........ 99
Strangury from Blisters ........... 540
Stroud on Physical Death of Christ.. 453
Surgeon, Malgaigne on Qualifications, 254
Swan on the Sympathetic Nerve .... 237
Sweat, Bloody, Stroud on.......... 457
Syphilis, Marchal on treatment of .. 57
Tænia, frequency in Algeria........
Tarsus, Dislocation of the.......... 148
Tendons, Painfnl Crepitation of .... 537
Todd's Physiological Anatomy ...... 326
Tommasini, Biographical Sketch of.. 271
Tongue, Papillæ of the ............ 328
Tonics in Acute Disease ........... 556
Tonsils, Extirpation of the ......... 51
Uterus, Glands of the ............ 11
Uterus, formation of the decidua of the 12
Uterus, Crosse on Inversion of the .. 124
Uterus, Blood-vessels of the ........ 280
Uterus, diseases of, after menstruation 387
Uterus, state of in Pregnancy ...... 391
Uterus, Inflammation of ...... 397, 404
Uterus, Gonorrhœal Inflammation of, 398
Uterus, Prolapsus of .............. 400
Uterus, Auscultation ofthe ........ 499
Vagina, Mucus of the ............ 378
Vertebra, Owen's Theory of the .... 160
Vertebrata, Skeletons of .......... 154
Vivisection, Macilwain on.......... 518
Vomiting, Seymour on treatment of.. 100
Vulva, inspection of .............. 531
Weber on Uterine Glands.......... 11
Weber on the Placenta ............ 20
Whitehead on Abortion and Sterility 377
Wounds, union of ................ 50

